# The Future of Uncertainty Factors With *In Vitro* Studies Using Human Cells

**DOI:** 10.1093/toxsci/kfab134

**Published:** 2021-11-10

**Authors:** Michael Dourson, Lorna Ewart, Suzanne C Fitzpatrick, Silvia B M Barros, Brinda Mahadevan, A Wallace Hayes

**Affiliations:** 1 TERA, Cincinnati, Ohio 45223, USA; 2 Emulate Inc., Boston, Massachusetts 02210, USA; 3 CFSAN, U.S. Food and Drug Administration, College Park, Maryland 20740, USA; 4 University of São Paulo, Sao Paulo, Brazil; 5 Brincor Associates, LLC, New Albany, Ohio 43054, USA; 6 College of Public Health, University of South Florida, Tampa, Florida 33612, USA

**Keywords:** alternatives, new approach methodologies, uncertainty factors, organ-on-a-chip

## Abstract

New approach methodologies (NAMs), including *in vitro* toxicology methods such as human cells from simple cell cultures to 3D and organ-on-a-chip models of human lung, intestine, liver, and other organs, are challenging the traditional “norm” of current regulatory risk assessments. Uncertainty Factors continue to be used by regulatory agencies to account for perceived deficits in toxicology data. With the expanded use of human cell NAMs, the question “Are uncertainty factors needed when human cells are used?” becomes a key topic in the development of 21st-century regulatory risk assessment. M.D., PhD, the coauthor of an article detailing uncertainty factors within the U.S. EPA, and L.E., PhD., Executive Vice President, Science, Emulate, who is involved in developing organ-on-a-chip models, debated the topic. One important outcome of the debate was that in the case of *in vitro* human cells on a chip, the interspecies (animal to human) uncertainty factor of 10 could be eliminated. However, in the case of the intraspecies (average human to sensitive human), the uncertainty factor of 10, additional toxicokinetic and/or toxicodynamic data or related information will be needed to reduce much less eliminate this factor. In the case of other currently used uncertainty factors, such as lowest observable adverse effect level to no-observed adverse effect level extrapolation, missing important toxicity studies, and acute/subchronic to chronic exposure extrapolation, additional data might be needed even when using *in vitro* human cells. Collaboration between traditional risk assessors with decades of experience with *in vivo* data and risk assessors working with modern technologies like organ chips is needed to find a way forward.

New approach methodologies (NAMs) include technologies, methodologies, approaches, or combination thereof that can provide information on chemical hazard and risk assessment that avoids testing with intact animals. Although NAMs are challenging the traditional “norm” of regulatory risk assessment that has been in place for many years, the U.S. Food and Drug Administration is fully supportive of developing, qualifying, and using NAMs as appropriate (https://www.fda.gov/science-research/about-science-research-fda/advancing-alternative-methods-fda; last accessed August 2021). NAMs, including *in vitro* toxicology methods using human cells, are available from simple cell cultures to 3D and organ-on-a-chip models of human lung, intestine, liver, and other organs.

The use of uncertainty factors by regulatory agencies to account for perceived deficits in toxicology data using animals led to the question at the center of the roundtable session at the 45th Annual Meeting of the Society of Toxicology “Are uncertainty factors needed when human cells are used?” The introduction of factors interchangeably referred to as safety, uncertainty, correction, assessment, adjustment, or extrapolation factors cannot be separated from the need to derive safe levels of additives or contaminants in food or other areas of regulatory toxicology. These factors originate from the emergence of the acceptable daily intake (ADI), a concept widely credited to European and American toxicologists including the late French toxicologist Professor Truhaut in the early 1950s (Hayes and Kruger, 2014). These uncertainty factors were introduced to extrapolate toxicological data from animal experiments to humans. A safety factor of 100 was originally proposed by Lehman and Fitzhugh (1954) of the U.S. Food and Drug Administration). A safety factor of 100 was arbitrarily set but was originally intended to account for interspecies (animal-to-human) variability and interindividual (human-to-human) variability, which allowed sensitive human populations to be compared with healthy experimental animals. The further subdivision of the conventional 10-fold safety factors for each experimental animal to human extrapolation and within human variability into toxicokinetics and toxicodynamics subfactors, was proposed independently by Renwick (1991) and the U.S. EPA (1994), and further expanded by Dorne and Renwick (2005).

For environmental chemicals, several additional factors beyond the initial 100 safety factor arbitrarily suggested by Lehman and Fitzhugh were proposed to account for various shortcomings in the experimental data, such as inappropriate study design, using acute or subchronic rather than chronic data (since the objective was to determine a lifetime ADI), or relying on a lowest observable adverse effect level (LOAEL) rather than a no-observed adverse effect level (NOAEL), as well as other factors such as severe or irreversible effects (Dourson and Stara, 1983). With the passage of the Food Quality Protection Act (FQPA) in 1996, an additional factor of 10 must be considered for children and other hypersensitive populations by the U.S. Environmental Protection Agency (U.S. EPA) unless the science supports otherwise [EPA/FQPA. Public Law 104–170, *Food Quality Protection Act*. Washington, DC: Office of the Federal Register, National Archives and Records Administration, U.S. Government Printing Office.]. The U.S. EPA (2002) has opined on how this factor is to be interpreted and used.

Dr M.D. initiated the debate by reminding us that uncertainty factors are considered necessary adjustments in the experimental animal *in vivo* dose showing no adverse effects to the expected *in vivo* no-effect dose for a sensitive subgroup of humans and that these factors will be needed even with studies using human cells *in vitro*. This no-effect dose in a sensitive subgroup is synonymous with an ADI or other similar safe or acceptable dose concepts, described in Figure  1 and more extensively by Dourson and Stara (1983), Dourson and DeRosa (1991), Dourson *et al.* (1996), Dourson and Parker (2007), and Dankovic *et al.* (2015).

**Figure 1.**
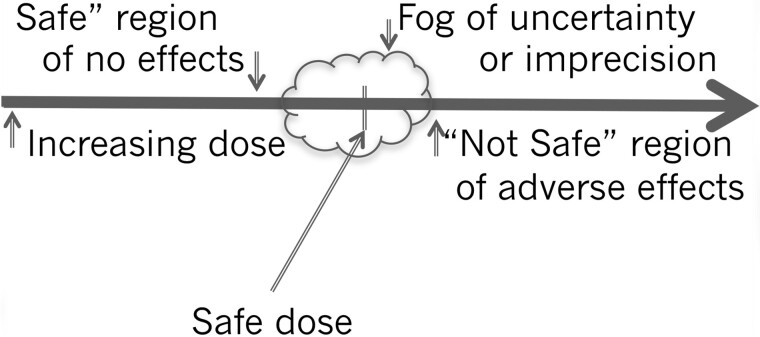
The safe dose concept.

In the development of safe doses with *in vivo* data, the use of different uncertainty factors for different chemicals is often necessary, because the underlying experimental data are not always uniform. The use of different uncertainty factors is currently a routine part of safe dose assessment because of these differing databases. Typical uncertainty factor used by the U.S. EPA is shown in Figure  2, where 2 of these factors are seen to reduce the projected risk (UF_H_ and UF_L_) and the other 3 factors are seen to move from 1 dose-response curve to another without any risk reduction. Other regulatory authorities often use a similar uncertainty factor construct.

**Figure 2.**
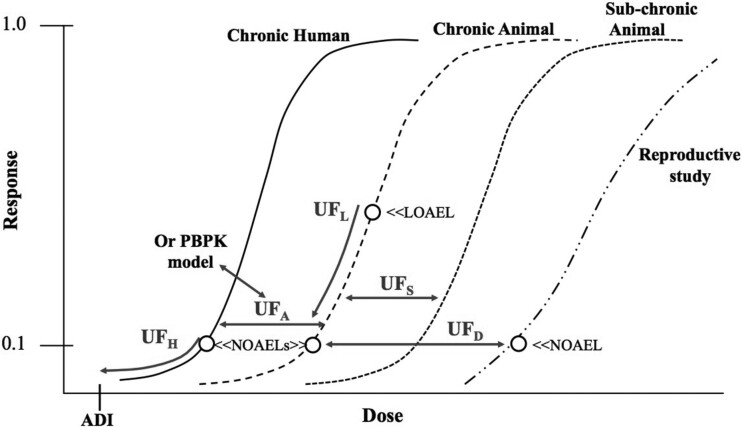
Areas of uncertainty to consider in non-cancer dose response assessment.

Although some consider uncertainty factors as arbitrary, this is a misconception as suggested by a quotation from a famous historical figure:

It is the mark of an instructed mind to rest satisfied with the degree of precision which the nature of the subject permits and not to seek an exactness where only an approximation of the truth is possible.

##  

### Aristotle

Rather than being arbitrary, uncertainty factors are imprecise. This is because the underlying biology is imprecise as readily demonstrated by innumerable biological measurements. Now if someone were to recommend the use of an uncertainty factor for the experimental animal to human extrapolation when starting with human data… that would be arbitrary!

Yet, another misconception of uncertainty factors, is that the default uncertainty factor for addressing human variability is too small.

- Supposition: Human variability in a toxic response is often well beyond 10-fold in response to drugs and unintended chemical exposures.
- Supposition: This variability is easily demonstrated in clinical trials, human observational studies, and in *in vitro* systems that use cells or organelles from different human populations.
- Therefore: The usual default uncertainty factor of 10-fold to estimate a safe dose from human data can be seen as not near enough in many cases.

Really? No, not really.

Human variability is indeed diverse, sometimes reaching hundreds and perhaps even thousands fold. But uncertainty factors, and specifically the one for human variability, never start with the most resistant individual, but rather from an individual or group in the more sensitive area of the dose-response curve as shown in Figure  3. Thus, the usual 10-fold uncertainty factor for this area of extrapolation accounts for larger variability in the human population, when used correctly.

**Figure 3.**
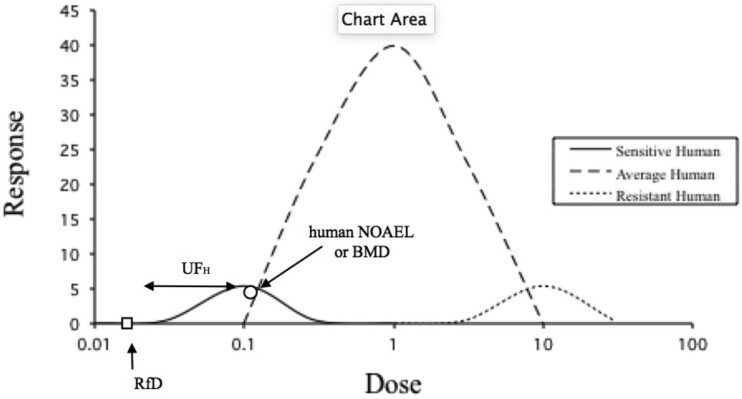
Hypothetical response as a function of dose for humans of different sensitivities.

So what about the future? The plethora of *in vitro* data from human cells will eliminate the need for at least one of the traditional factors… that of experimental animal to human extrapolation. But like *in vivo* data, *in vitro* databases may also not likely be uniform amongst chemicals. Thus, uncertainty factors for human variability, subchronic to chronic exposure, and LOAEL to NOAEL, will most certainly be needed.

Furthermore, additional uncertainties can be envisioned. For example, uncertainties in determining the critical effect of a chemical will also increase with *in vitro* data, since not all interorgan, or even intraorgan, interactions are testable *in vitro*. In addition, one of the more common critical effects is the loss of body weight. Do we have an organ chip for that? Well, perhaps not yet.

Moreover, the use of such *in vitro* data will introduce additional uncertainties, not even ones traditionally considered, such as extrapolation from *in vitro* concentration to *in vivo* exposure.

Insofar as *in vitro* data can address these concerns, fewer uncertainty factors might be needed in the future. We look forward to working with colleagues to incorporate these new data into future risk assessments.

Dr L.E. countered by asserting that advanced *in vitro* technologies can reduce the need for uncertainty or safety factors. Toxicology is not new. Indeed, it can be traced back to the work of the physician Paracelsus in the 15th century. And what is interesting about this infamous quote from Paracelsus is that the concept of dose, and thus exposure, was central to the determination of response. Now in the 21st century, exposure assessment remains central to the risk assessment paradigm, but decades of research have also pointed to the need to integrate these data with that identifying the hazard and the characterization of the hazard. Given the advances in metrology, we can now measure substances and their subsequent response at increasingly greater degrees of sensitivity. As such detailed databases can be built that contribute to the overall risk assessment process. However, even with advances in science and technology, hazard assessment remains typically based on data from animal models which is then extrapolated to humans, a process most toxicologists agree is subject to degrees of uncertainty. But, the human population is also more diverse than the average inbred laboratory animal and with data from deeper genetic profiling together with knowledge on the role environmental factors can play, further uncertainty creeps into the risk assessment paradigm when considering the impact of diversity. At the end of the day risk assessment is a conservative endeavor although in today’s modern world it is warranted to consider alternative or complementary approaches to achieve the same overall goal.

So, how can greater certainty be brought to risk assessment? Toxicologists have long since recognized that there are many steps between the “dose” of a substance and the resultant toxic response. By breaking this big step down into a series of smaller ones, we can start to discover potential opportunities for increasing certainty and potentially reducing the need for arbitrary safety factors. These smaller steps involve assessment of the target organ dose rather than a total body dose, consideration of target organ metabolism, and then accounting for different organ sensitivities to a toxic substance. Organ-Chip technology, a developing field that combines engineering with cell biology, provides an advanced *in vitro* culture system that faithfully recreates cellular microenvironments enabling cells to retain their physiological functionality (Bhatia and Ingber, 2014). By emulating biochemical and biophysical factors that drive cellular behavior, resultant data should more readily translate to *in vivo* outcomes. Central to Organ-Chip models is the recreation of the tissue-tissue interface which is enabled by culturing organ-specific cells in 2 independent, parallel microfluidic channels (Figure  4). The optimized cell culture medium then flows in a unidirectional fashion and can be collected at multiple time points for the analysis of biochemical markers. Uniquely, drugs or chemicals can be added to either channel thus allowing the study of exposure and effect at the physiologically relevant point of contact (Chou *et al.*, 2020). Organ-Chips are also designed to faithfully recreate the physical microenvironment either through the shear stress as a consequence of dynamic flow or when cultured on flexible substances, mechanical forces can be also be applied to replicate key organ motions such as breathing in the lung (Huh *et al.*, 2010; Hassell *et al.*, 2017) or peristalsis in the intestine (Apostolou *et al.*, 2021). Atmospheric oxygen can also be altered to enable the coculture of complex bacterial consortia (Jalili-Firoozinezhad *et al.*, 2019). Thus, the technology may be able to create greater certainty, reducing the need for uncertainty factors.

**Figure 4.**
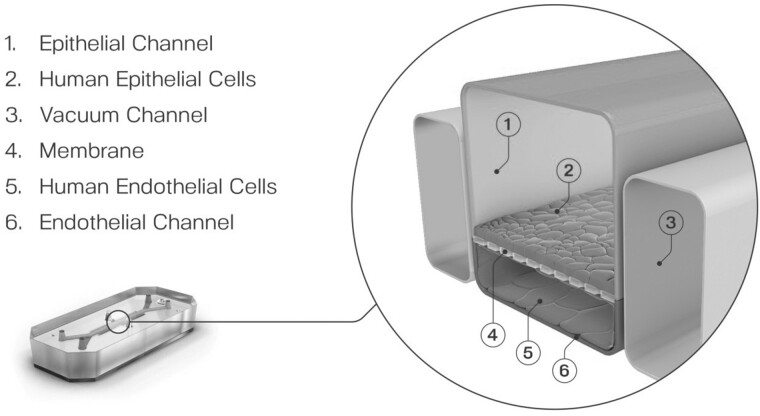
Central to Organ-Chip models is the recreation of the tissue-tissue interface which is enabled by culturing organ-specific cells in 2 independent, parallel microfluidic channels.

Ultimately exposure to a toxic substance occurs through ingestion, respiration, or direct contact with the skin before distribution throughout the body. Organ chips can enable a better approximation of absorption and thus can enable the determination of the target organ dose, the first step toward increasing certainty during risk assessment. This was nicely illustrated in the landmark lung-on-a-chip paper published in Huh *et al.* (2010) at Harvard’s Wyss Institute. In this study, Huh and colleagues recreated the alveolus-air interface using human alveolar epithelial cells and human pulmonary microvascular endothelial cells and simulated breathing motion by applying a vacuum stretch. Ultrafine nanoparticles were applied to the epithelial compartment to induce pulmonary inflammation and the resultant cellular oxidative stress was analyzed by reactive oxygen species (ROS) imaging. Application of nanoparticles without strain showed a small increase in ROS production compared with control but in the presence of 10% strain, ROS generation was significantly greater. By providing a more physiological microenvironment, the true toxic potential of the nanoparticles at the point of contact was revealed. Furthermore, in the presence of strain, there was a greater translocation of the nanoparticles into the vascular compartment of the chip thus enabling investigation of distribution to other organs. The same type of study could be done with skin or intestine chips.

Once it is known what could be distributed through the vasculature and therefore reach other organs, target organ chips can then be used to determine if the dose is in the relevant toxic range for that organ. Importantly, this can be determined under the conditions in which the toxicant must pass through the endothelial-parenchymal tissue interface as would happen *in vivo* and such a determination is lacking in most other *in vitro* models. So, the next question is can different target organ chips demonstrate differential sensitivity to the same toxic substance? Exposure to 4–8 gray ionizing gamma-radiation, whether therapeutic or accidental, may result in acute radiation syndrome that is associated with gastrointestinal disturbances including abdominal pain, diarrhea, and vomiting, and when left untreated, it can result in intestinal hemorrhage, sepsis, and death. Using the intestinal cell line Caco2 together with intestinal endothelial cells, the effect of radiation exposure has been assessed in organ chips (Jalili-Firoozinezhad *et al.*, 2018). By measuring villi height, it was shown that chips exposed to radiation resulted in a blunting of the villi whereas this response was lessened in the presence of the medical countermeasure dimethyloxaloyl glycine (DMOG) that showed a degree of protection with respect to villi height. Moreover, radiation exposure also significantly impacted the tight junctional protein ZO-1 which would contribute to a reduction in the intestinal barrier. The disruption of the barrier was demonstrated using labeled dextran to assess barrier permeability. Over 72 h, radiation exposure caused an increase in apparent permeability indicating increased barrier permeability integrity whereas chips also treated with DMOG retained the barrier integrity. Taking this a step further, the potential mechanism of action was assessed with TUNEL staining. The authors found within the first 24 h of exposure to radiation the endothelial cells were sensitive to the radiation but by 48 h, epithelial cells also showed an increase in the number of TUNEL positive cells. DMOG treatment reduced the number of TUNEL positive cells. Furthermore, ROS formation was measured which was increased in the presence of radiation whereas it was reduced in the presence of DMOG. Taken together, the chips point toward an apoptotic cell death via ROS production. These rich mechanistic data are essential to enhance risk assessment.

Bone marrow is another target organ for gamma radiation with sublethal doses resulting in bone marrow suppression leading to immunosuppression in exposed individuals. Work published by Chou *et al.* (2020) looked at 2 doses of radiation in a human bone marrow chip and the effect of this on myeloid or erythroid cells. There was a concentration-dependent reduction in myeloid and erythroid cells. Additionally, by profiling 2 different populations of each cell type, Chou *et al.* (2020) illustrated that the less mature cells (eg, CD16 lo or E1) were more susceptible to the effects of radiation. This is interesting because these are likely to be the cells that are proliferating. Overall, the team demonstrated that the bone marrow was sensitive to much smaller amounts of radiation (2 gray) compared with the intestine. In summary, human organ chips accurately reflect the differential response of human organs to gamma radiation, offering evidence that safety factors may not be required to determine safe levels of exposure.

Most *in vitro* models involve bathing cells in a medium containing drug (or toxicant) which is not representative of the *in vivo* response. This is 1 reason why animal models are favored in hazard characterization because the toxicokinetics can be measured. Owing to the microfluidics, exposure dynamics can be recreated in organ chips offering a further advantage to their use in the risk assessment paradigm. Staying with the bone marrow chip, Chou *et al.* (2020) were able to exemplify how organ chips can reproduce clinical exposure profiles and how these profiles are connected to the clinical response. AZD2811, a drug in development within AstraZeneca’s oncology therapy area, was assessed within the bone marrow chip. As is common with oncology therapeutics, the bone marrow is a target organ for toxicity. In clinical trials, patients received a 2-h infusion of AZD2811 which resulted in anemia (Boss *et al.*, 2011), and a reformulation of the drug tested in a subsequent clinical trial over a 48-h infusion resulted in neutropenia (Schwartz *et al.*, 2013). The human bone marrow chip was able to reproduce this complex concentration-effect-time relationship. Furthermore, by measuring the impact on the myeloid and erythroid populations, the bone marrow chip reproduced the clinical responses that were reported. Taken together, data such as these illustrate the value that organ chips can bring to human risk assessment.

Since extrapolation between species introduces uncertainty into the risk assessment process, can organ chip technology address this uncertainty? Because organ chips are agnostic to cell sources, cells from nonhuman animals and humans can be seeded within the chip enabling scientists to get closer to the goal of predicting human response using surrogate models that show concordance. Jang *et al.* (2019) created liver chips using human, rat, or dog cells. It was shown that each of the species liver chips maintained albumin and urea production over 14 days in culture with the production of these markers being higher, and within the expected physiological range, compared with conventional cell culture. Because the metabolism of xenobiotics and other toxic substances is a key function of the liver, the study also demonstrated that liver chips with human, rat, or dog cells displayed activity across 3 major P450 families, CYP1A, 2B, and 3A. In all cases, the chip also outperformed the conventional 2D sandwich and metabolic competency was maintained in liver chips for 14 days. In some cases, this competency was comparable to activity seen in freshly isolated hepatocytes. These data provide evidence that chips can not only be used to measure functionality in nonclinical species but that organ chips can also contribute to the acquisition of data to understand the effects that target organ metabolism can have on the assessment of risk. Finally, this seminal work also showed that the species liver chips were able to discriminate species differences to the endothelin receptor antagonist Bosentan. Bosentan inhibits the bile salt export pump (BSEP) which is involved in the elimination of bile salts from the hepatocyte. Inhibition of BSEP results in accumulation of bile salts which drives a cholestatic liver injury. The accumulation of bile salts was measured in chips using the fluorescent molecule cholyl-lysyl-fluorescein which is a substrate for BSEP. In the presence of Bosentan, there was greater fluorescence confirming transporter inhibition. Humans and dogs are sensitive to the effects of Bosentan on the BSEP transporter whereas rats are not. By measuring albumin production and calculating IC_50s_, it was shown that human chips were the most sensitive to Bosentan, with the hepatotoxicity occurring at an *in vivo* relevant dose. This concordance between nonhuman animals and animals *in vitro* and *in vivo* data sets increases the confidence that organ chip technology can predict animal and human response.

Finally, how can organ chips represent human population diversity? This is arguably the greatest challenge and will require careful selection of multiple cell donors, but it remains to be determined how many donors are needed to give confidence that population variability is being correctly represented. For the development of Chemical-Specific Adjustment Factors, the IPCS (International Programme on Chemical Safety) (2005) suggests that the number of subjects within the population, or within the major subgroup if there are 2 or more groups, should be sufficient to provide an accurate measure of the central tendency. As a guide, the standard error (SD of the sample divided by the square root of the sample size) should be less than approximately 20% of the mean. Based on available data, this would normally involve a minimum number of approximately 5 subjects or samples from 5 individuals, unless the variability is very low (ie, small coefficient of variability).

Both presenters provided a short rebuttal that following their original arguments before the Q&A session. Two panel members, Dr S.B.M.B. and Dr B.M. put forth interesting questions. Dr S.B.M.B.’s question was addressed to Dr L.E.: *How can chronic in vivo exposure studies be modeled on organ chips?* Dr L.E. explained that cellular functionality has been studied for up to 28 days on organ chips. However, the chronicity question can only be answered through a combination of 28 days of good, solid data and building a mathematical model around that information to understand chronic exposure.

Dr B.M. question was addressed to Dr M.D.: *Considering that animal data are obtained from high-dose studies and then moving on to low doses, how can this complement what is done on organ chips*? Dr M.D. explained that the advantage of *in vitro* systems is that mechanisms of toxicity are better understood *in vitro* than *in vivo*, that there are limitations of observing critical effects *in vivo*, and it may be that *in vitro* data can provide answers in such cases. Additionally, mixture assessments can be assessed a lot quicker through *in vitro* systems than through *in vivo* approaches.

One important outcome of the workshop debate was that in the case of *in vitro* human cells on a chip, the interspecies (animal to human) uncertainty factor of 10 could be eliminated. However, in the case of the intraspecies (average human to sensitive human), the uncertainty factor of 10, additional toxicokinetic and/or toxicodynamic data or related information will be needed to reduce much less eliminate this factor. In the case of other currently used uncertainty factors, such as LOAEL to NOAEL extrapolation, missing important toxicity studies, and acute/subchronic to chronic exposure extrapolation, additional data might be needed even when using *in vitro* human cells. The Roundtable session concluded with the panel members agreeing that collaboration is needed between traditional risk assessors with decades of experience with *in vivo* data and risk assessors working with modern technologies like organ chips to find a way forward. 

## DECLARATION OF CONFLICTING INTERESTS

The authors declared no potential conflicts of interest with respect to the research, authorship, and/or publication of this article. 
